# A Magnetically Controlled Capsule Robot with Biopsy Capability for Intestinal Applications

**DOI:** 10.3390/s25237158

**Published:** 2025-11-24

**Authors:** Lingling Zheng, Jie Sun, Zhengdong Qi, Le Yan, Fen Zhao, Haifei Zhang, Le Zhang, Zixu Wang, Shuxiang Guo

**Affiliations:** 1 School of Information Engineering, Nanjing Xiaozhuang University, Nanjing 211171, China; 2 School of Artificial Intelligence, Nanjing Xiaozhuang University, Nanjing 211171, China; 3 Department of Electronic and Electrical Engineering, Southern University of Science and Technology, Shenzhen 518055, China

**Keywords:** capsule endoscopy, capsule robot, Intestine, magnetically controlled capsule robot, magnetic actuation, biopsy

## Abstract

Magnetically controlled capsule robots offer unique advantages for performing intestinal biopsies. In this paper, we propose a novel Magnetically Controlled Capsule Robot for Intestinal Biopsy (MCCR-IB), capable of both navigation and biopsy within the intestine. To address the coupling issue between locomotion control and biopsy control, a magnetic field locking method is also introduced. The locomotion performance, curved-passage capability, and biopsy ability were experimentally evaluated. Under a 7 Hz rotating magnetic field, the MCCR-IB achieved forward and backward velocities of 20.22 mm/s and 18.27 mm/s, respectively, with a biopsy needle puncture force of 1.99 N. Furthermore, ex vivo experiments were conducted to preliminarily verify the feasibility of the robot’s motion and biopsy functions. The experimental results demonstrate that the proposed MCCR-IB exhibits good performance and shows promising potential for future clinical applications.

## 1. Introduction

Minimally invasive diagnosis and therapy have become a major trend in gastrointestinal medicine due to their advantages of reduced trauma, enhanced safety, and improved patient comfort [1]. The capsule endoscope, as an important derivative of robotic technology, enables noninvasive examination of the gastrointestinal tract through oral ingestion [2,3,4]. Although capsule endoscopy has made significant progress in imaging-based diagnosis, its functionality remains limited to passive imaging, without the ability to perform biopsies or pathological analysis [2]. Consequently, the development of biopsy capsule robots has attracted increasing research attention in recent years [5,6,7,8,9,10].

The actuation methods of capsule robots can generally be categorized into passive and active types. Passive capsule robots rely on natural intestinal peristalsis to move through the gastrointestinal tract, lacking the capability for active locomotion or targeted navigation [11,12,13]. In contrast, active capsule robots are equipped with internal actuation mechanisms that enable autonomous movement, allowing them to reach desired locations for targeted diagnosis or therapeutic interventions [14,15]. Common actuation approaches include pneumatic actuation [16], motor-driven actuation [17,18], and magnetic actuation [19,20,21]. Among these, magnetic actuation has emerged as the mainstream technique for active capsule robots owing to its wireless controllability and compact structure. Zhang et al. proposed a novel magnetically actuated capsule robot equipped with two internal permanent magnets for oscillatory motion and anchoring, as well as a shape memory alloy-driven actuator for biopsy sampling [22]. The robot underwent safety, locomotion performance, and functional evaluation experiments, demonstrating advantages in driving capability and overall functionality. Rashid et al. developed a capsule robot containing two internal permanent magnets, which is actuated by an external electromagnetic driving system to control both the capsule’s locomotion and the biopsy mechanism [23]. A spring-loaded structure was integrated into the biopsy mechanism to enable the retraction of the biopsy tool after tissue sampling. Xu et al. proposed a multifunctional capsule-shaped puncture biopsy robot capable of performing tissue sampling, thermal hemostasis, and multi-stage drug delivery [24]. In [25], a biopsy capsule robot based on a negative-pressure suction principle was developed for liquid sampling in the gastrointestinal tract. The capsule robot adopts a magnetic spring structure, in which the suction port can be aligned with the target sampling area by controlling the direction of the external magnetic field. When the external magnetic field is strengthened, the magnetic spring is triggered to generate negative pressure, thereby enabling liquid sampling. Sitti et al. proposed a magnetically actuated soft capsule robot that utilizes fine-needle biopsy technology to collect tissue samples from deep gastric layers [3]. The fine needle is controlled by a magnetic gradient field, which induces deformation of the soft robot, thereby extruding the needle for tissue penetration.

For the control of biopsy puncture, recent studies have primarily utilized magnetic gradient forces to manipulate the biopsy needle [3,26]. However, the absence of a force-amplifying mechanism results in a relatively low puncture force [3]. Increasing the magnetic field strength could enhance the puncture capability but would also impose higher demands on the magnetic field generation system, leading to increased cost and complexity [6]. Some other studies have employed rotating magnetic fields to generate torque for controlling the puncture motion [5,23]. Nevertheless, when both locomotion and biopsy operations are driven by the same magnetic actuation system, coupling between locomotion control and biopsy control easily occurs [23]. Moreover, since the biopsy needle is typically located at the front end rather than on the lateral side of the capsule, it becomes difficult to access intestinal tissues effectively [5,23]. Even when tissue samples are obtained, the sampling direction is lateral, resulting in a limited biopsy depth and making it challenging to collect deep-layer tissue specimens.

To address the aforementioned challenges, we propose a novel Magnetically Controlled Capsule Robot for Intestinal Biopsy (MCCR-IB), which utilizes a rotating magnetic field to achieve both locomotion and biopsy operations. To mitigate the coupling between locomotion control and biopsy control, a magnetic field locking method is also introduced. By applying a gradient magnetic field, the main body of the robot can be “locked” in position, allowing independent control of the biopsy mechanism.

The remainder of this paper is organized as follows. Section 2 introduces the overall system architecture and the design of the MCCR-IB, followed by the control strategy and force analysis of the robot under magnetic actuation, as well as an analysis of its capability to traverse curved intestinal pathways. Section 3 presents experimental evaluations of the locomotion performance, curved-passage capability, and biopsy capability, along with detailed discussions. Preliminary ex vivo experiments are also conducted to validate the feasibility of the MCCR-IB. Finally, Section 4 concludes the study.

## 2. Materials and Methods

### 2.1. Robot System

**Figure 1:**
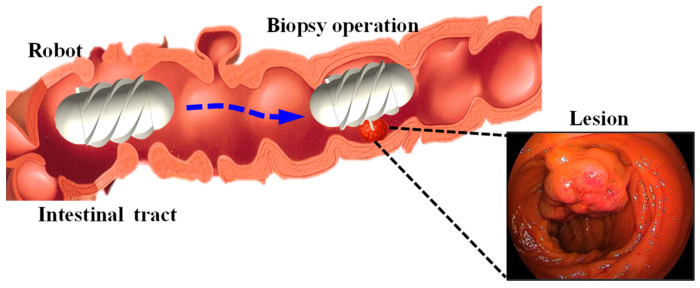
Illustration of the intestine containing a lesion and the biopsy procedure performed by the magnetically controlled capsule robot.

**Figure 2:**
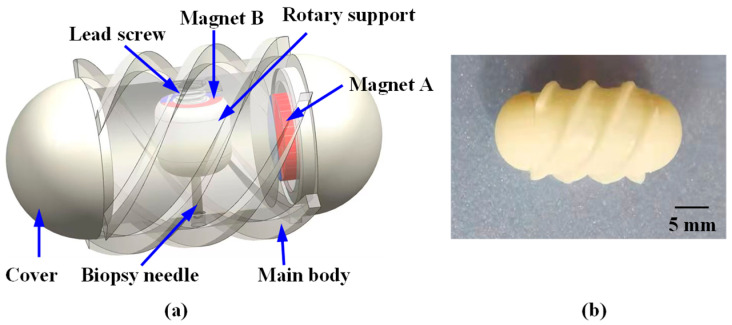
Structural design and prototype of the proposed Magnetically Controlled Capsule Robot for Intestinal Biopsy (MCCR-IB). (**a**) Structural diagram; (**b**) Prototype of the MCCR-IB. For all magnets, the magnetization direction is illustrated from the red (north) pole toward the blue (south) pole. All magnets exhibit radial magnetization directions.

Figure 1 illustrates the motion of the robot within the intestine containing a lesion, where it reaches the target position to perform a biopsy. To achieve this function, the proposed system consists of the MCCR-IB and a magnetic field generation system for controlling the MCCR-IB. The magnetic field generation system is located outside the human body and produces the required magnetic field to control the MCCR-IB inside the body. The structural design of the MCCR-IB is shown in Figure 2a. The MCCR-IB contains two permanent magnets: Magnet A, used for locomotion, actuates the robot inside the intestine; and Magnet B, used for biopsy, controls the biopsy mechanism to perform tissue sampling. Magnet A is rigidly connected to the main body, with its magnetization oriented radially. The outer surface of the main body is equipped with helical threads, enabling the robot to move forward or backward. Both ends of the main body are sealed with two covers. Magnet B is fixed to a rotary support, which can rotate around a lead screw to generate linear motion. A biopsy needle is mounted on the rotary support. The linear motion of the rotary support drives the biopsy needle to extend from the main body to collect intestinal tissue. After sampling, the rotary support reverses its rotation, retracting the biopsy needle back into the main body. In this study, the MCCR-IB was fabricated using 3D printing, as shown in Figure 2b. The overall dimensions of the MCCR-IB are 14 mm in diameter and 29 mm in length.

**Figure 3:**
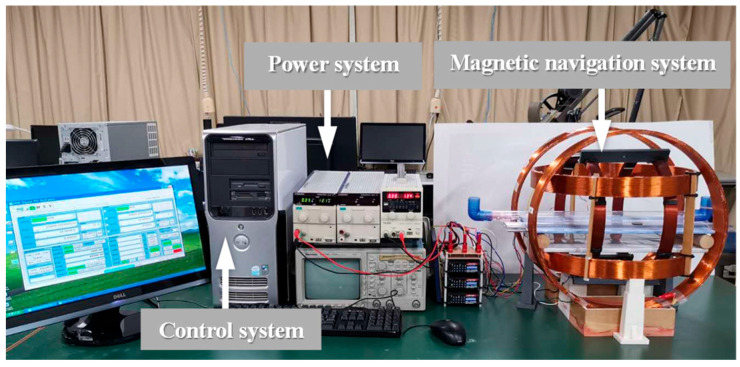
Magnetic field generation system for controlling the proposed MCCR-IB.

To generate the desired magnetic field, a magnetic field generation system was developed, as illustrated in Figure 3. The system consists of three main components: a control system, a power system, and a magnetic navigation system. The control system produces command signals, which are transmitted through the power system to drive the magnetic navigation system. This process generates the corresponding magnetic fields required to control the MCCR-IB. The magnetic navigation system comprises six coils, arranged in three orthogonal pairs, each capable of producing a magnetic field along one of the *x*, *y*, and *z* axes.

### 2.2. Robot Control

**Figure 4:**
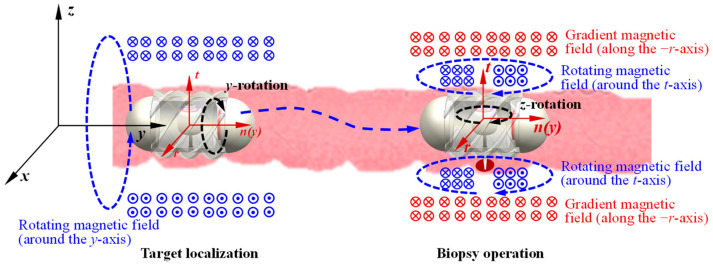
Flowchart of the control and operation process of the MCCR-IB. The xyz coordinate system represents the global reference frame, while the rtn coordinate system denotes the frame moving with the robot. The circles with a dot or a cross at the center indicate the direction of the magnetic flux lines. For all magnets, the magnetization direction is illustrated from the red (north) pole toward the blue (south) pole (also shown in Figure 2). All magnets exhibit radial magnetization directions.

The biopsy operation using the MCCR-IB within the intestine involves two types of motion: target localization and biopsy operation. During target localization, the robot moves forward or backward inside the intestine to reach the desired target position. As illustrated in Figure 4, an external rotating magnetic field is applied around the *y*-axis. Under this rotating field, the MCCR-IB moves toward the target position and eventually reaches it. In this process, Magnet A rotates in response to the applied magnetic field, providing the locomotion of the robot, while Magnet B experiences only a minor magnetic torque, resulting in slight oscillation. The detailed mechanics analysis and discussion are presented in Section 2.3.

When the MCCR-IB reaches the target position, the external magnetic field is switched to a rotating magnetic field around the *z*-axis. In this state, the field primarily actuates Magnet B, driving the motion of the biopsy needle mounted on the rotary support to perform tissue sampling within the intestine. During this process, Magnet A experiences only slight magnetic perturbations, allowing the robot to maintain a stable position at the lesion site without interfering with the biopsy procedure. The detailed mechanics analysis and discussion are presented in Section 2.3. During the biopsy operation, an action–reaction force exists between the rotary support and the main body, which induces a counter-rotation of the MCCR-IB along the *z*-axis. However, this rotation is physically constrained by the intestinal wall, preventing noticeable displacement of the robot. Consequently, this mechanism enables reliable and stable execution of the biopsy.

In practical applications, it is challenging to apply a precisely rotating magnetic field strictly around the *y*- or *z*-axis. During target localization, when a rotating magnetic field is applied around the *y*-axis, a slight angular deviation may occur. As a result, not only does Magnet A experience a torque for locomotion, but Magnet B also receives a small unintended torque. This minor torque can drive the rotary support, potentially causing undesired motion of the biopsy needle. To address this issue, the rotary support in our design incorporates an initial threshold torque. When the applied torque is smaller than this threshold, the rotary support remains stationary; only when the torque exceeds the threshold does the rotary support begin to rotate. This approach effectively prevents unintended motion of the biopsy needle during the locomotion phase of the robot.

Similarly, during the biopsy operation, when a rotating magnetic field is applied around the *z*-axis, a certain deviation angle may occur, causing the robot to rotate correspondingly. To address this issue, we propose a magnetic field locking method. This method introduces a gradient magnetic field during the biopsy process to constrain the robot’s rotation. As shown in Figure 4, a gradient magnetic field is applied along the −*r*-axis (−*x*-axis). Under this gradient magnetic field, Magnet A experiences a force directed along the −*r*-axis (−*x*-axis), which pulls the robot toward the intestinal wall (closer to the biopsy site). At this time, a rotating magnetic field is applied along the *t*-axis (*z*-axis) to drive Magnet B for performing the biopsy. Because the gradient magnetic field presses the robot against the intestinal wall, together with the effects of intestinal folds, a counteracting torque is generated by the surrounding tissue. This torque suppresses the rotation induced by the rotating magnetic field, thereby achieving rotational locking of the robot. The gradient magnetic field must be oriented radially toward the biopsy site to ensure that the robot is pressed precisely against the target region. Since this gradient magnetic field does not induce rotation of Magnet A, it does not affect the robot’s locomotion. Likewise, it does not rotate Magnet B, and therefore does not interfere with the biopsy actuation.

### 2.3. Mechanical Analysis

As illustrated in Figure 4, the robot performs two functions, locomotion and biopsy—each driven by a distinct magnetic field. For analytical convenience, two coordinate systems are defined: the *xyz* coordinate system represents the global reference frame, which is fixed to the ground, while the *rnt* coordinate system represents the robot reference frame, which moves together with the robot. The origin of the *rnt* frame is located at the geometric center of the robot, with the *n*-axis aligned with the *y*-axis of the global frame and the *t*-axis coinciding with the axis of the rotary support. Both the *xyz* and rnt coordinate systems are established as right-handed coordinate systems. The following sections analyze the locomotion and biopsy mechanisms of the robot, respectively.

During the locomotion process, as illustrated in Figure 4, the external magnetic field rotates around the *y*-axis and can be expressed as

(1) $$B\left(t\right)=B_{0}\left(\cos\theta \;\hat{x}+\sin\theta \;\hat{z}\right)$$

where $B_{0}$ denotes the amplitude of the magnetic field, θ is the phase angle of the field, and $\hat{x}\;$ and $\hat{z}$ represent the unit vectors along the *x*- and *z*-axes, respectively. Transforming the magnetic field from the global coordinate system (*xyz*) to the robot coordinate system (*rnt*) yields

(2) $$B\left(t\right)=B_{0}\left(\cos\sigma \;\hat{r}+\sin\sigma \;\hat{t}\right)$$

where σ denotes the phase difference between the magnetic field and the robot, expressed as

(3) $$\sigma =\theta -\theta_{A}$$

with $\theta_{A}$ representing the rotation angle of the robot about its own longitudinal axis. The torque acting on Magnet A can be determined by

(4) $$T_{A}=m_{A}\times B$$

where $m_{A}$ the magnetic moment of Magnet A. According to Equation (4), the torque components acting on Magnet A around the *x*- and *z*-axes are zero, and the torque around the y-axis is given by

(5) $$T_{A-y}=m_{A}B_{0}\sin\sigma$$

Magnet B is also subjected to the magnetic torque produced by the same rotating field. Following a similar analytical procedure, the torque can be expressed in the robot coordinate system. Because the magnetic field and the magnetization direction of Magnet B are not aligned, the resulting torque can be decomposed into three directional components, expressed as

(6) $$T_{B-n}=-m_{B}B_{0}\cos\left(\theta_{B}+\beta_{0}\right)\cos\sigma$$

(7) $$T_{B-t}=-m_{B}B_{0}\sin\left(\theta_{B}+\beta_{0}\right)\sin\sigma$$

(8) $$T_{B-r}=m_{B}B_{0}\sin\left(\theta_{B}+\beta_{0}\right)\cos\sigma$$

where the torque components acting on Magnet B in the robot coordinate system can be expressed as $T_{B-n}$, $T_{B-r}$, $T_{B-t}$, representing the torques about the *n*-, *r*-, and *t*-axes, respectively; $m_{B}$ denotes the magnetic moment of Magnet B; $\theta_{B}$ is the rotation angle of the rotary support (Magnet B) about its own axis; $\beta_{0}$ represents the initial angle between the magnetization direction of Magnet B and the *r*-axis of the robot coordinate system.

During locomotion, the robot is simultaneously subjected to the magnetic torques generated by Magnet A and Magnet B. In the direction of motion, the total torque around the *y*-axis can be expressed as

(9) $$T_{total-y}=T_{A-y}+T_{B-n}$$

Accordingly, the rotational dynamics of the robot can be described as

(10) $$J_{A}\ddot{\theta_{A}}+c_{A}\dot{\theta_{A}}+\tau_{rA}=m_{A}B_{0}\sin\sigma -m_{B}B_{0}\cos\left(\theta_{B}+\beta_{0}\right)\cos\sigma$$

where $J_{A}$ denotes the moment of inertia of the robot, $\;c_{A}$ is the viscous damping coefficient, and $\tau_{rA}$ epresents the external load torque acting on the robot. Similarly, the rotational motion of the rotary support can be expressed as

(11) $$J_{B}\ddot{\theta_{B}}+c_{B}\dot{\theta_{B}}+\tau_{rB}=-m_{B}B_{0}\sin\left(\theta_{B}+\beta_{0}\right)\sin\sigma$$

where $J_{B}$ is the moment of inertia of the rotary support (including the biopsy needle), $\;c_{B}$ is its damping coefficient, and $\tau_{rB}$ is the external load torque acting on the rotary support.

During the locomotion process, the torque acting about the *y*-axis—originating from both Magnet A and Magnet B—governs the robot’s rotational motion, as described by Equations (9) and (10). The torques in other directions, which arise from the reaction torques of Magnet B (as defined in Equations (7) and (8)), are counteracted by the intestinal wall. Consequently, these components do not affect the overall locomotion of the robot. During this process, the rotary support experiences a torque about its own axis (*t*-axis), which tends to induce rotation, as described in Equation (11). However, since the torque is proportional to sinσ—a zero-mean alternating term—it does not result in continuous rotation of the rotary support but only causes small oscillatory motion. Such oscillation does not drive the biopsy needle to extend outside the main body and therefore does not interfere with the operation. To further improve system stability and reliability, the external load torque $\tau_{rB}$ can be appropriately increased to suppress these minor oscillations.

The above analysis describes the magnetic actuation and dynamic behavior of the robot under a rotating magnetic field applied around the *y*-axis during locomotion. During the biopsy operation, a rotating magnetic field is instead applied around the *z*-axis. Since the analytical process is analogous, it is not further discussed here.

### 2.4. Curved-Passage Capability

When the robot moves through a curved intestinal tract, both the inner diameter and the curvature of the intestine significantly affect its locomotion performance. If either the intestinal diameter or the curvature radius is smaller than a certain critical threshold, the robot is unable to move through the intestine.

**Figure 5:**
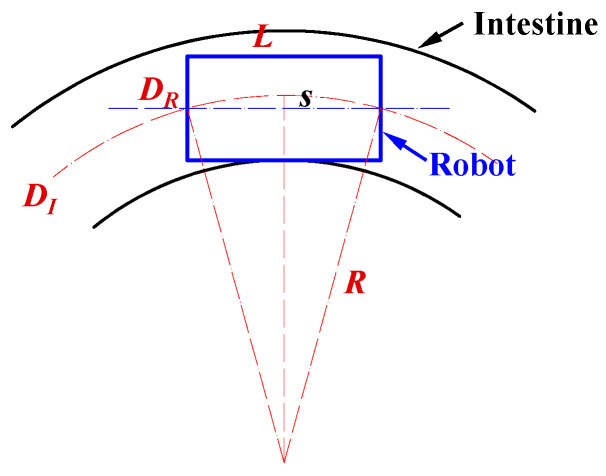
Analysis of the robot’s maneuverability in curved intestinal segments. In the diagram, $D_{R}$ is the robot diameter, L means the robot length, $D_{I}$ indicates the intestinal inner diameter, and R is the curvature radius of the intestine.

To analyze the robot’s passability in curved sections, the motion is simplified, and a geometric model is established, as illustrated in Figure 5. In this model, both the robot and the intestinal wall are treated as rigid bodies. The robot is modeled as a cylinder with an outer diameter $D_{R}$ and length L; the intestinal tract is represented by a cylindrical channel with an inner diameter $D_{I}$ and curvature radius R. The clearance between the robot and the intestinal wall can be expressed as:

(12) $$\delta \;=\frac{D_{I}-\;D_{R}}{2}$$

As shown in Figure 5, the sagitta corresponding to a chord length L can be expressed as

(13) $$s=R-\sqrt{R^{2}-{\left(\frac{L}{2}\right)}^{2}}$$

A necessary geometric condition for the robot to pass through the curved intestinal section without physical interference is that the sagitta does not exceed the available radial clearance, that is

(14) s ≤ δ 

By combining Equations (13) and (14) and solving for the lower bound of the curvature radius R, the following relationship is obtained:

(15) $$R\;\geq \;\frac{L^{2}}{8\delta }+\frac{\delta }{2}$$

Substituting Equation (12) into Equation (15) yields

(16) $$R\;\geq \frac{L^{2}}{4\left(D_{I}-D_{R}\right)}+\frac{D_{I}-D_{R}}{4}$$

Equation (16) reveals the geometric relationship among the robot diameter $D_{R}$, robot length L, intestinal inner diameter $D_{I}$, and curvature radius R. Based on this relationship, the theoretical correlation among these parameters was plotted, as shown in the figure in Section 3.2. In addition, the experimental results presented in Section 3.2 were incorporated into the same figure to compare the theoretical and experimental values. These results provide valuable theoretical guidance for the design and practical application of the magnetic capsule robot in intestinal biopsy.

## 3. Performance Verification

### 3.1. Locomotion

#### 3.1.1. Experimental Setup

**Figure 6:**
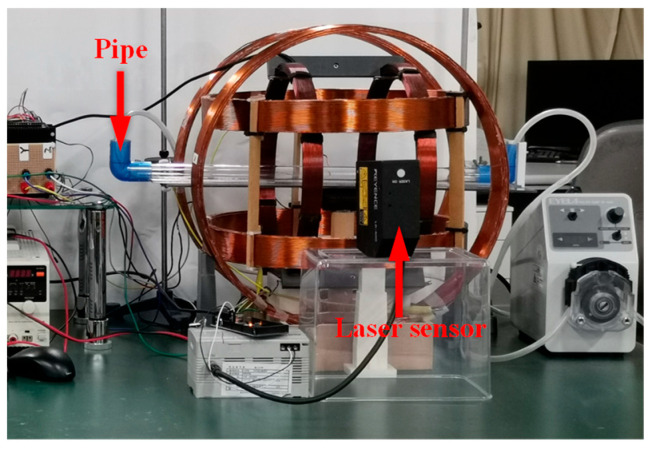
Experimental setup for the locomotion test.

To evaluate the locomotion feasibility of the MCCR-IB under magnetic actuation, a series of locomotion experiments were conducted. The experimental setup is illustrated in Figure 6. A pipe with an inner diameter of 31 mm was placed inside the magnetic navigation system to serve as the motion channel for the robot. To measure the robot’s translational velocity, a laser displacement sensor (LK-500, KEYENCE, Osaka, Japan) was positioned alongside the magnetic navigation system. The control system generated command signals to produce a rotating magnetic field along the pipe’s longitudinal axis. The magnetic field frequency was increased from 0 to 10 Hz in 1 Hz increments. The robot was driven forward under each frequency condition, and its velocity was recorded using the laser displacement sensor. Each test was repeated three times for averaging. Subsequently, the rotation direction of the magnetic field was reversed to drive the robot backward, and the backward velocity was measured in the same manner.

**Figure 7:**
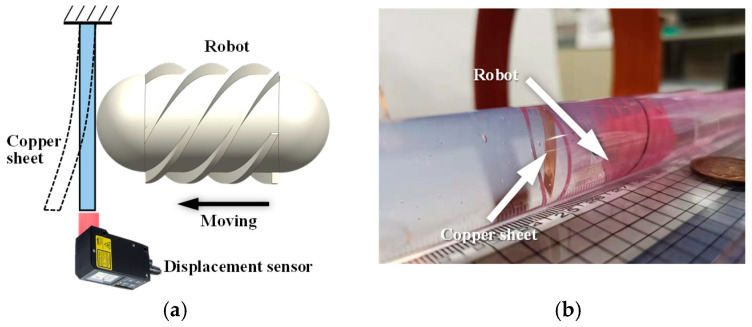
Experimental setup for measuring the propulsion force of the robot. (**a**) Schematic diagram: the robot moves toward the copper sheet fixed at the distal end and induces bending deformation of the sheet; (**b**) photograph of the experimental setup.

In addition, the propulsion force of the robot was experimentally evaluated. The setup is shown in Figure 7. For convenience of measurement, a copper sheet was fixed inside the pipe. During locomotion, the robot pushed the copper sheet, causing measurable deformation. The copper sheet was modeled as a cantilever beam, and the propulsion force of the robot was calculated based on the deformation of the beam. Both forward and backward motions were tested three times, respectively.

#### 3.1.2. Experimental Results and Discussion

**Figure 8:**
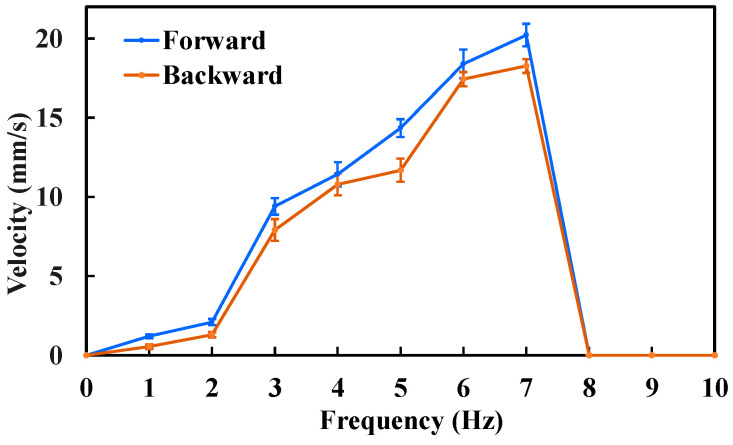
Locomotion velocity of the MCCR-IB under an external magnetic field with various frequencies. Each type of test was repeated 3 times (*n* = 3). Points represent mean values; error bars denote the standard deviation.

The forward and backward velocities of the MCCR-IB under different magnetic field frequencies are shown in Figure 8. As observed, the robot’s locomotion speed increases with the magnetic field frequency. This trend occurs because a higher rotating magnetic field frequency induces a faster rotation of the robot, thereby generating a higher translational velocity. The proposed MCCR-IB achieved a maximum forward velocity of 20.22 mm/s and a maximum backward velocity of 18.27 mm/s. It was also observed that when the magnetic field frequency exceeded 7 Hz, both the forward and backward velocities dropped to zero. During locomotion, the robot’s rotation follows the external magnetic field with a certain phase lag. As the magnetic field frequency increases, this phase difference also increases. When the phase difference exceeds a critical threshold, the magnetic torque acting on the robot decreases sharply, preventing further rotation.

**Figure 9:**
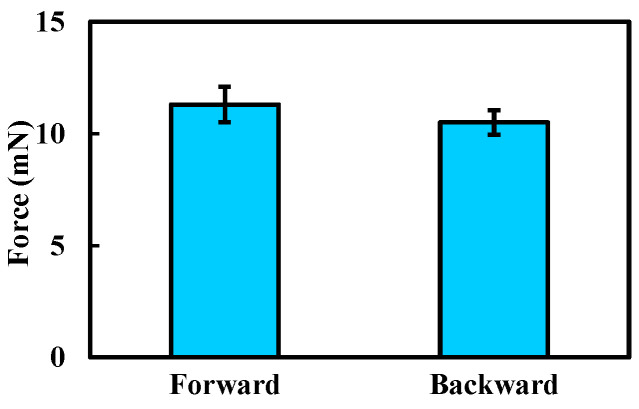
Experimental results of the propulsion force measurement. Each type of test was repeated 3 times (*n* = 3). Bars represent mean values; error bars denote the standard deviation.

Based on the results shown in Figure 8, the maximum locomotion speed was achieved at a magnetic field frequency of 7 Hz, which was therefore selected for the propulsion force measurement. The experimental results are presented in Figure 9. The maximum propulsion forces during forward and backward motion were 11.3 mN and 10.5 mN, respectively. The difference in propulsion force again arises from the asymmetric structural design of the MCCR-IB. The propulsion force of the robot is primarily influenced by three factors: the magnetic field strength, the magnetization intensity of Magnet A, and the thread geometry on the main body’s surface. Increasing either the magnetic field strength or the magnetization intensity can effectively enhance the propulsion capability. In practical applications, these parameters can be adjusted to adapt the robot’s performance to specific clinical environments.

### 3.2. Curved-Passage Capability Test

#### 3.2.1. Experimental Setup

**Figure 10:**
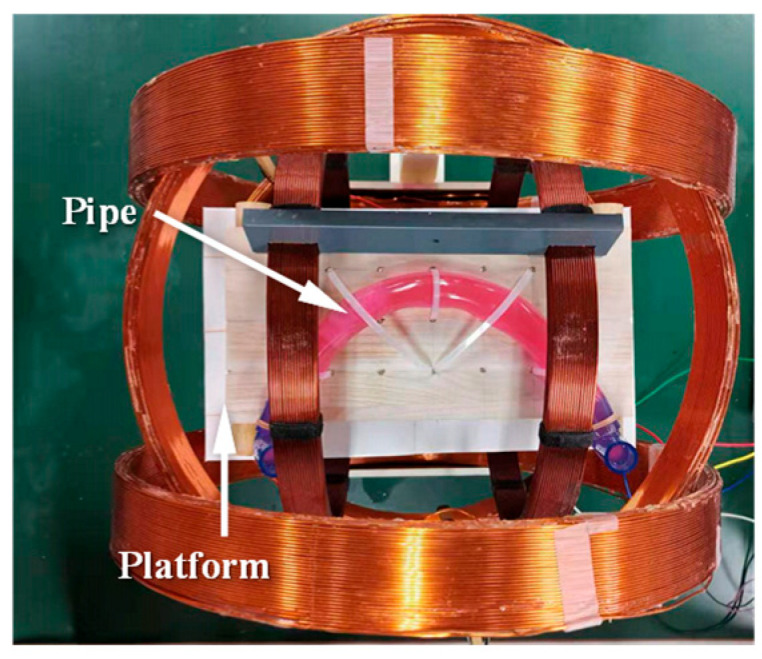
Experimental setup for testing the curved-passage capability of the MCCR-IB.

To evaluate the curved-passage capability of the MCCR-IB, an experimental setup was designed as illustrated in Figure 10. A flexible pipe was fixed onto a platform to simulate the curved intestinal environment. Flexible pipes with various inner diameters were used, ranging from 15 mm to 27 mm in 2 mm increments. For each flexible pipe, different curvature radii were configured to form curved sections through which the robot was driven. The experiment was conducted to determine whether the robot could successfully traverse each configuration. For a given inner diameter, the curvature radius was gradually increased until the robot was able to pass through the curved section. The corresponding inner diameter and curvature radius at which successful passage occurred were recorded.

#### 3.2.2. Experimental Results and Discussion

In Section 2.4, the curved-passage capability of the robot was theoretically analyzed.

**Figure 11:**
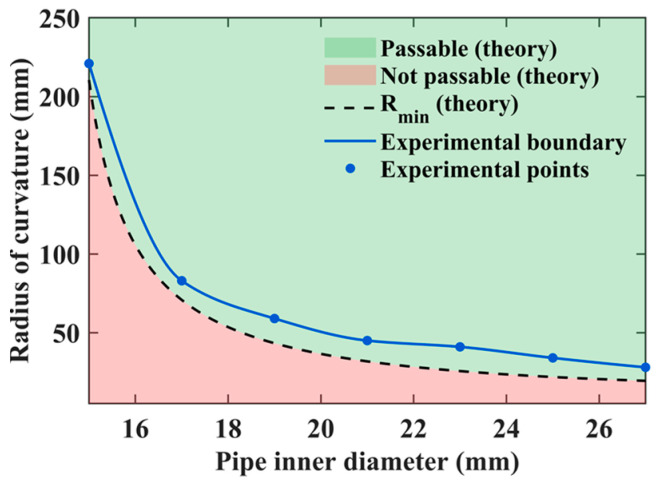
Comparison between theoretical and experimental results of the robot’s curved-passage capability.

To visualize this capability more intuitively, we conducted theoretical calculations by varying both the inner diameter and the curvature radius of the pipe. According to the National Cancer Institute (NCI) [27], the small intestine is a tube measuring about 25 mm in diameter. The proposed robot has a length of 29 mm, and thus, we set the pipe’s inner diameter range to 15–27 mm. Regarding the curvature radius, to the best of our knowledge, no clinical data are currently available. Thus, we conservatively set the initial value to 5 mm and gradually increased it until the robot could pass completely. Since a curvature radius of 5 mm is unrealistically small for the intestine, this conservative assumption provides a meaningful reference. The theoretical relationship was plotted, as shown in Figure 11, where the horizontal and vertical axes represent the pipe’s inner diameter and the curvature radius of the bend, respectively. In the figure, the light green region indicates the passable region, while the light red region corresponds to the non-passable (infeasible) region. The black dashed line represents the theoretical boundary.

Experimental tests were conducted to validate the theoretical results, and the measured data points were superimposed on Figure 11. The blue dots indicate the experimentally obtained data points, which were connected by a curve to form the experimental boundary line. This boundary represents the actual parameter conditions under which the robot can successfully pass through curved sections in practice.

It was observed that the experimental boundary (blue curve) does not coincide with the theoretical one (black dashed line). This discrepancy arises because the real environment is more complex than the idealized theoretical model, imposing stricter conditions for successful passage. For example, when the pipe’s inner diameter was 19 mm, the theoretical curvature radius required for passage was 44.65 mm, whereas in experiments, the robot could only pass when the curvature radius reached 59 mm. Similarly, for a curvature radius of 41 mm, the theoretical minimum pipe diameter was 19.3 mm, while the experimental result indicated that a diameter of 23 mm was necessary for successful passage. These theoretical and experimental results together provide important guidance for the design optimization and practical application of the MCCR-IB in curved intestinal environments.

### 3.3. Biopsy Performance

#### 3.3.1. Experimental Setup

To evaluate the biopsy performance of the proposed MCCR-IB, three types of experiments were conducted. First, the motion velocity of the biopsy needle was measured to assess the biopsy efficiency. Second, the puncture force generated during the biopsy process was measured to evaluate the feasibility of tissue acquisition. Finally, the oscillation angle under the magnetic field was measured to evaluate its controllability.

For the biopsy efficiency test, lead screws with different pitches were used, ranging from 0.25 mm to 1.5 mm with an increment of 0.25 mm. The corresponding rotary supports were assembled accordingly. Under a rotating magnetic field, the laser displacement sensor (as shown in Figure 6) was employed to measure the linear velocity of the biopsy needle. The frequency of the rotating magnetic field was set to 1 Hz, 3 Hz, and 5 Hz, respectively. For each combination of lead-screw pitch and magnetic field frequency, the needle velocity was measured three times and averaged.

**Figure 12:**
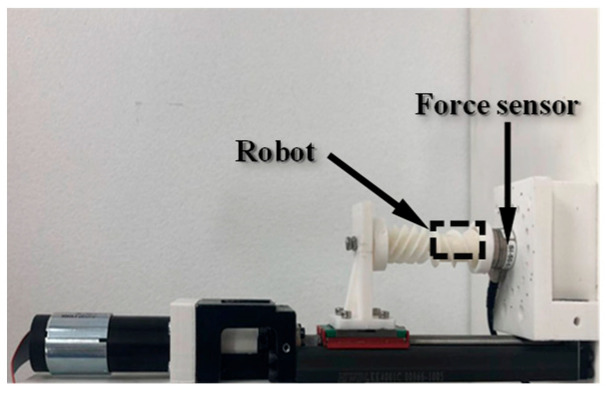
Experimental setup for measuring the biopsy puncture force.

For the puncture force measurement, an ATI force sensor was used to record the axial force exerted at the tip of the biopsy needle, as shown in Figure 12. A rotating magnetic field with a frequency of 5 Hz was applied, with its rotation axis aligned with the biopsy needle. Similar to the biopsy efficiency test, lead screws with pitches ranging from 0.25 mm to 1.5 mm (incremented by 0.25 mm) were used, and the corresponding rotary supports were assembled. Each experiment was repeated three times for consistency.

In the third experiment, to evaluate the locking effect of the robot under different gradient magnetic fields, gradients ranging from 0 mT/m to 60 mT/m were applied in increments of 20 mT/m. The robot’s oscillation behavior was observed, and the corresponding deviation angle was measured. Each experimental condition was tested three times.

#### 3.3.2. Experimental Results and Discussion

**Figure 13:**
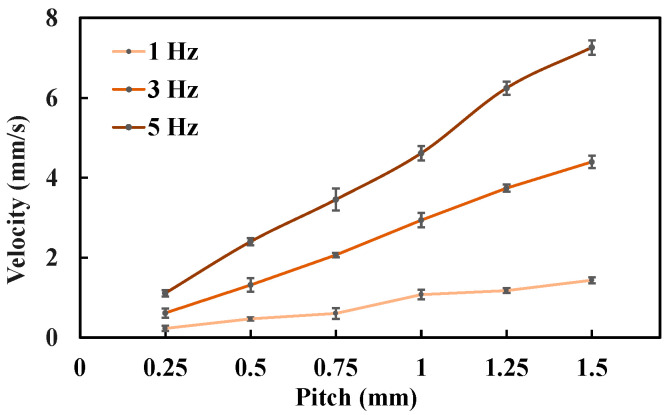
Biopsy puncture velocity under different experimental conditions. Each type of test was repeated 3 times (*n* = 3). Points represent mean values; error bars denote the standard deviation.

The biopsy puncture velocity under different conditions is shown in Figure 13. As the lead-screw pitch increases, the biopsy needle velocity correspondingly increases. Under a 3 Hz rotating magnetic field, the biopsy needle velocity was 1.32 mm/s when the lead-screw pitch was 0.5 mm, and it increased to 3.74 mm/s when the pitch was 1.25 mm. This occurs because, under the same magnetic field frequency, a larger lead-screw pitch results in a greater linear displacement per revolution of the rotary support. Similarly, the biopsy needle velocity also increases with the rotating magnetic field frequency. For example, when the lead-screw pitch was 1.0 mm, the biopsy needle velocity increased from 1.08 mm/s at 1 Hz to 4.61 mm/s at 5 Hz. A higher magnetic field frequency drives the rotary support to rotate faster, thereby generating greater linear displacement of the biopsy needle.

**Figure 14:**
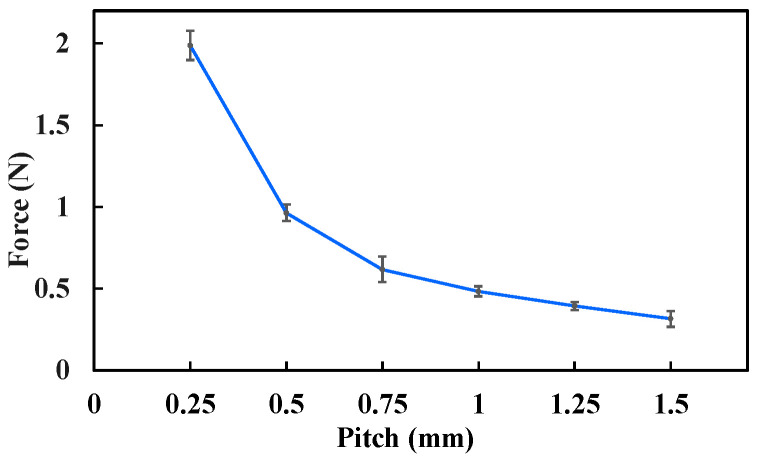
Biopsy puncture force under different experimental conditions. Each type of test was repeated 3 times (*n* = 3). Points represent mean values; error bars denote the standard deviation.

The measured biopsy puncture forces under different conditions are presented in Figure 14. The results show that the puncture force decreases as the lead-screw pitch increases. Specifically, when the pitch was 0.5 mm, the puncture force was 0.96 N; when the pitch increased to 1.5 mm, the puncture force decreased to 0.32 N. This reduction occurs because a larger lead-screw pitch corresponds to a larger lead angle, which reduces the axial component of the transmitted force, resulting in a smaller puncture force.

By comparing Figure 13 and Figure 14, it can be observed that the biopsy velocity and puncture force exhibit an inverse relationship. This trend is consistent with the principle of constant input energy—as one increases, the other decreases. Therefore, when selecting the lead-screw pitch, both the biopsy velocity and the puncture force should be considered comprehensively. In practice, since the puncture force is more critical to ensure biopsy success, it is often preferable to sacrifice velocity to maintain sufficient puncture capability. Furthermore, the puncture force also depends on the magnetic field strength and the magnetization intensity of Magnet B. Increasing either of these parameters can effectively enhance the biopsy puncture force, providing greater flexibility for adapting the MCCR-IB to different clinical tissue environments.

**Figure 15:**
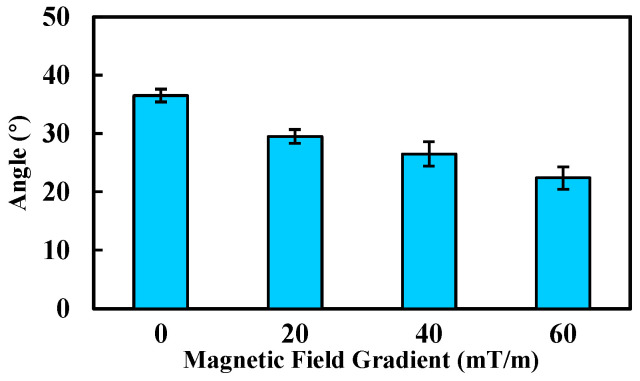
Deviation angle of the robot under various gradient magnetic fields. Each type of test was repeated 3 times (*n* = 3). Bars represent mean values; error bars denote the standard deviation.

The deviation angle of the MCCR-IB under different gradient magnetic fields is shown in Figure 15. As the magnetic field gradient increased from 0 mT/m to 60 mT/m, the deviation angle decreased markedly from 36.5° to 22.4°. A clear reduction in deviation amplitude was observed with increasing gradient strength. In addition, a stronger gradient field would further reduce the oscillation. These results demonstrate the feasibility and effectiveness of the proposed method.

### 3.4. Ex Vivo Experiment

#### 3.4.1. Experimental Setup

To further verify the feasibility of the proposed MCCR-IB, an ex vivo experiment was conducted. A porcine small intestine was used to simulate the human intestinal environment. The intestine samples were obtained from a commercial food supplier, and therefore no ethical concerns were involved.

**Figure 16:**
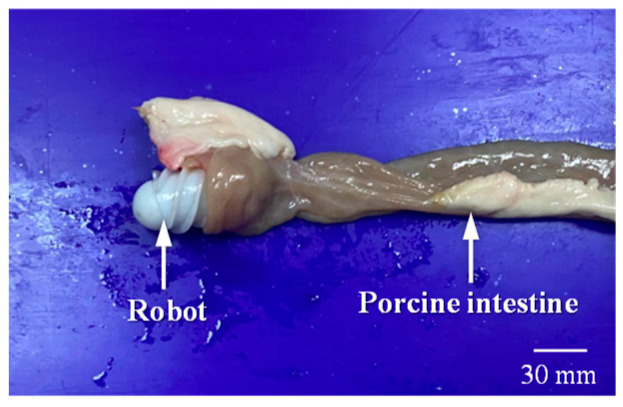
The MCCR-IB and porcine intestine used in the ex vivo experiment.

**Figure 17:**
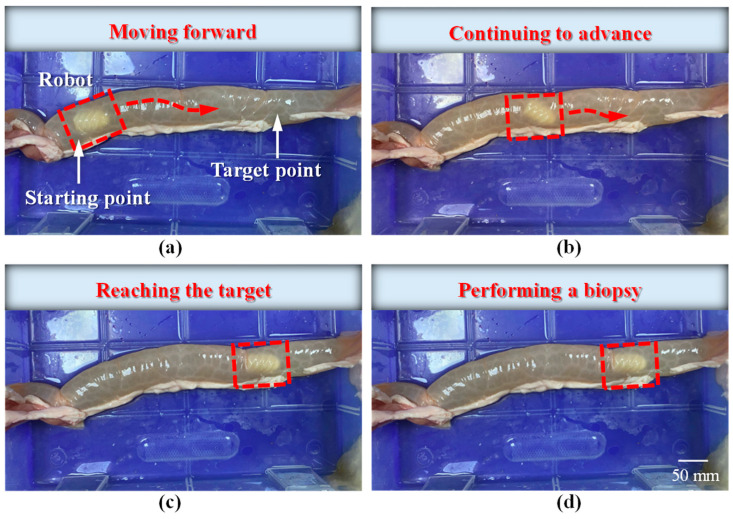
Locomotion of the robot inside the intestine: (**a**) the robot starts moving from the initial position toward the target; (**b**) the robot continues moving forward; (**c**) the robot reaches the target position; (**d**) the robot performs the biopsy operation.

As shown in Figure 16, the MCCR-IB was inserted from one end of the porcine intestine, and water was injected into the lumen to maintain a hydrated environment. As illustrated in Figure 17, two positions were defined within the intestine: a starting position and a target position. The robot began locomotion from the starting position and moved forward along the intestinal lumen. Upon reaching the target position, the robot stopped and performed the biopsy operation. The entire locomotion and biopsy process were recorded, and after completion, the tip of the biopsy needle was examined to confirm whether intestinal tissue had been successfully collected.

#### 3.4.2. Experimental Results and Discussion

**Figure 18:**
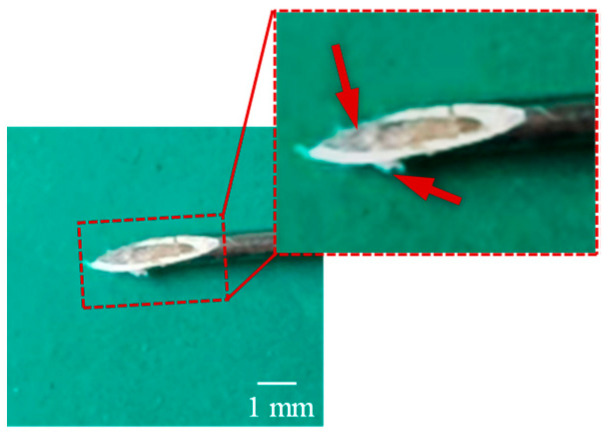
Intestinal tissue sample collected by the biopsy needle after the ex vivo experiment.

The locomotion process of the MCCR-IB inside the porcine intestine is illustrated in Figure 17. In Figure 17a, the MCCR-IB begins locomotion from the starting position. In Figure 17b, it moves toward the target position through the middle section of the intestine. In Figure 17c, the robot reaches the target position and stops. Finally, in Figure 17d, the MCCR-IB performs the biopsy operation on the intestinal wall. These sequential images clearly demonstrate that the proposed MCCR-IB successfully achieved locomotion and biopsy inside the porcine intestine. Figure 18 shows the tissue sample extracted by the biopsy needle after the biopsy procedure. A visible amount of intestinal tissue adhered to the needle tip, confirming the feasibility of real-time intestinal biopsy using the proposed MCCR-IB.

In medical diagnostics, certain pathological analyses require a minimum tissue volume for accurate examination. In this study, the diameter of the biopsy needle was 0.7 mm. To increase the amount of collected tissue, the needle diameter can be enlarged accordingly. Furthermore, the sampling efficiency can be improved by adding barbs at the needle tip or applying negative pressure inside the biopsy needle, both of which enhance tissue retention and facilitate more effective specimen collection.

## 4. Conclusions

In this study, a novel biopsy capsule robot was proposed to achieve both locomotion and biopsy functions within the intestine. To address the coupling problem between locomotion control and biopsy control, a magnetic field locking method was also introduced. Laboratory tests and ex vivo experiments were conducted, and the results demonstrated that the proposed robot has potential for clinical applications. However, several limitations remain in this study. First, the prototype was fabricated using 3D printing with photosensitive resin, which is not biocompatible and therefore unsuitable for direct clinical use. In future work, biocompatible materials will be employed, and corresponding parameter adjustments and performance tests will be required due to material differences. Second, the propulsion force and puncture performance of the robot currently lack support from clinical data. Further studies are needed to optimize these parameters based on clinical requirements. Finally, in vivo and clinical experiments have not yet been performed. The experimental conditions used in this study differ from the actual clinical environment, and thus, the results may not fully reflect the robot’s performance in practical applications. In future research, we will address these limitations to further advance the clinical translation of the proposed biopsy capsule robot.

## Data Availability

The original contributions presented in this study are included in the article. Further inquiries can be directed to the corresponding authors.

## Abbreviation

The following abbreviation is used in this manuscript:

MCCR-IB	Magnetically Controlled Capsule Robot for Intestinal Biopsy
